# Safe diagnostic management of malignant mediastinal tumors in the presence of respiratory distress: a 10-year experience

**DOI:** 10.1186/s12887-020-02183-w

**Published:** 2020-06-10

**Authors:** Tomoko Tanaka, Hizuru Amano, Yujiro Tanaka, Yoshiyuki Takahashi, Tatsuro Tajiri, Takahisa Tainaka, Chiyoe Shirota, Wataru Sumida, Kazuki Yokota, Satoshi Makita, Yukiko Tani, Akinari Hinoki, Hiroo Uchida

**Affiliations:** 1 Department of Pediatric Surgery, Nagoya University Graduate School of Medicine, 65 Tsurumai, Showa, Nagoya, 466-8550 Japan; 2 Department of Pediatric Surgery, Graduate School of Medicine, The University of Tokyo, Tokyo, Japan; 3 Department of Pediatrics, Nagoya University Graduate School of Medicine, Nagoya, Japan; 4 Department of Pediatric Surgery, Kyoto Prefectural University of Medicine, Kyoto, Japan

**Keywords:** Malignant mediastinal tumor, Tracheal compression, Respiratory distress, Respiratory collapse

## Abstract

**Background:**

The fundamental treatment for patients with pediatric malignant mediastinal tumors is chemotherapy. Therefore, accurate diagnosis is essential for selecting the appropriate chemotherapeutic regimen. However, malignant mediastinal tumors occasionally cause respiratory distress, and biopsies under general anesthesia are dangerous for such patients as invasive mechanical ventilation can aggravate airway obstruction caused by mass effect. In this study, we reviewed our 10-year diagnostic experience to evaluate the efficacy of our practices and confirm a safe diagnostic protocol for future patients.

**Methods:**

We retrospectively reviewed medical records of children with malignant mediastinal tumors diagnosed at Nagoya University Hospital from 2007 to 2018 who demonstrated respiratory distress. Respiratory distress included dyspnea, massive pleural effusion, wheezing, and hypoxemia owing to tumors. Data on sex, age at onset, primary symptoms, location of tumor, management strategy (especially the method of diagnosis and definitive diagnosis), clinical course, prognosis during the acute phase (within 3 months from the onset of respiratory symptoms), and long-term outcome were collected.

**Results:**

Twelve pediatric patients met the review criteria. There were seven anterior mediastinal tumors and five posterior mediastinal tumors. All anterior mediastinal tumors were diagnosed via bone marrow smear, thoracentesis, or core needle biopsy while maintaining spontaneous breathing. Regarding posterior tumors, two patients were diagnosed via a core needle biopsy and lymph node excisional biopsy under spontaneous breathing. Two cases were initially diagnosed solely using tumor markers. One patient with severe tracheal compression underwent tumor resection with extracorporeal membrane oxygenation stand-by. No patient died of diagnostic procedure-related complications.

**Conclusions:**

In 11 of the 12 cases reviewed, safe and accurate tumor diagnosis was accomplished without general anesthesia. A diagnostic strategy without general anesthesia considering the tumor location proved to be useful.

## Background

Malignant mediastinal tumors in children include neuroblastoma, lymphoma, acute lymphoblastic leukemia, Langerhans histiocytosis, and germ cell tumor, among others. The primary treatment for these pediatric malignant mediastinal tumors is chemotherapy. Therefore, an accurate diagnosis is indispensable for selecting the appropriate chemotherapeutic regimen. Although not an intrinsically common complication, these mediastinal tumors can occasionally cause respiratory distress. In patients with respiratory distress whose bronchus is compressed by a mediastinal tumor, respiratory failure is likely to occur, especially when muscle-relaxant drugs are used for general anesthesia or mechanical ventilation [1]. Therefore, avoiding tumor biopsies for mediastinal tumors under general anesthesia has been recommended; the use of alternative diagnostic methods has also been advocated [2].

In our hospital, we have historically avoided diagnostic biopsy under general anesthesia for pediatric patients with malignant mediastinal tumors with respiratory distress. To assess the safety and efficacy of our diagnostic strategy and establish a safe diagnostic protocol for future pediatric patients with malignant mediastinal tumor causing some degree of respiratory distress, we conducted a thorough review of our previous 10-year diagnostic and treatment experience.

## Methods

This study protocol was approved by Nagoya University Hospital Institutional review board (2019–0089). Because this was a retrospective observational study and the data analyzed were anonymized, informed consent from participants or their parents/guardians was obtained through an opt-out method on our hospital website in accordance with the Ethical Guidelines for Medical and Health Research Involving Human Subjects in Japan. The medical records of all children diagnosed with malignant mediastinal tumor with respiratory distress at Nagoya University Hospital, Japan from January 2007 to December 2018 were retrospectively reviewed. Dyspnea, massive pleural effusion, wheezing, and hypoxemia owing to mediastinal tumors were considered to indicate respiratory distress. Data on sex, age at onset, primary symptoms, location of tumor, management strategy (especially the method of diagnosis and definitive diagnosis), clinical course, prognosis during the acute phase (within 3 months from the onset of respiratory symptoms), and long-term outcome were also reviewed.

## Results

During the study period, 44 pediatric patients with mediastinal tumor required surgical intervention at the department of pediatric surgery at our institution; in 24 patients, the tumor was malignant. Of these 24 patients with malignant mediastinal tumors, 12 patients (6 boys and 6 girls) had respiratory distress. The median age at onset was 59 months (interquartile range 5.8–112.3 months). There were four stage 3 non-Hodgkin lymphoma, three stage 3 neuroblastomas, two acute lymphocytic leukemias, and three other tumors (malignant rhabdoid tumor, Langerhans histiocytosis, and stage 4 SMARCA4-deficient thoracic sarcoma). Of these 12 tumors, 7 were located in the anterior mediastinum and 5 in the posterior mediastinum.

### Anterior mediastinal tumors

**Fig. 1:**
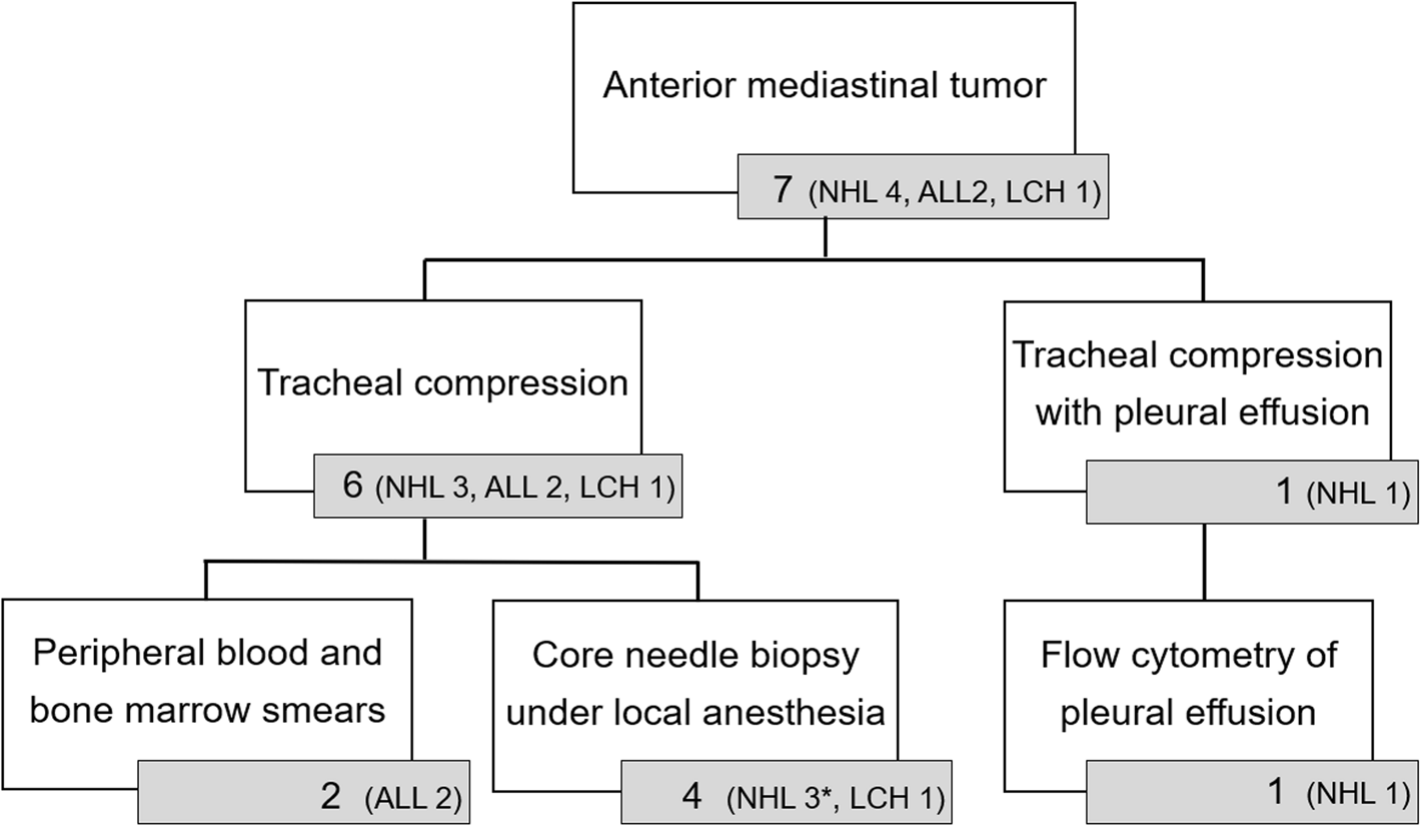
Diagnostic procedure for anterior mediastinal tumors with respiratory distress. NHL; non-Hodgkin lymphoma. ALL; acute lymphoblastic leukemia. LCH; Langerhans histiocytosis. *; biopsy was performed after chemotherapy in one case

**Fig. 2:**
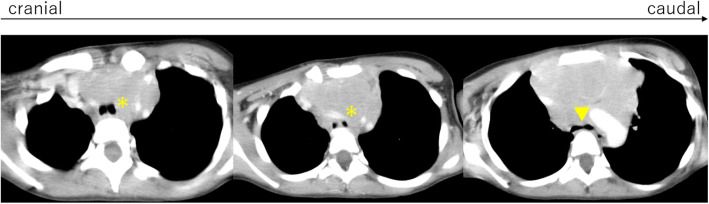
Computed tomography images of a patient who underwent chemotherapy without a biopsy. The trachea was severely compressed by the anterior mediastinal tumor (non-Hodgkin lymphoma). *; tracheal compression by the tumor at the trachea. ▼; tracheal bifurcation

Among the seven anterior mediastinal tumors, four were non-Hodgkin lymphoma, two acute lymphoblastic leukemias, and one Langerhans histiocytosis (Fig. 1). All seven tumors caused tracheal compression, and one also caused pleural effusion. Regarding the tracheal compression, three patients had < 50% compression, with initial symptoms of wheezing, general fatigue, and chest pain. The others had ≥50% compression, with initial symptoms of coughing, fever, wheezing, and dyspnea. Of the six patients without pleural effusion, three were diagnosed by core needle biopsy under local anesthesia and two were diagnosed based on the findings of peripheral blood and bone marrow smear examinations. The remaining patient whose trachea was severely compressed by the tumor, received chemotherapy and was diagnosed by core needle biopsy under local anesthesia while receiving chemotherapy (Fig. 2). The patient with pleural effusion was diagnosed based on findings of cytology and flow cytometry of the pleural effusion. All patients achieved tumor regression and survived the acute phase. One patient with stage 3 non-Hodgkin lymphoma died 5 months after the onset because chemotherapy was not effective.

### Posterior mediastinal tumors

**Fig. 3:**
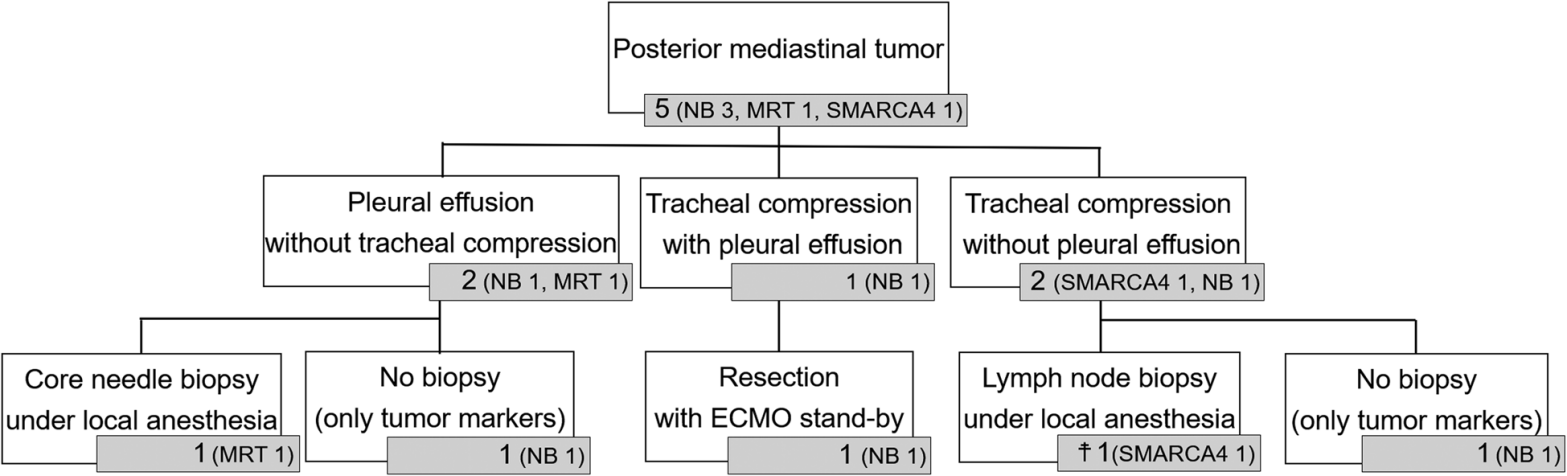
Diagnostic procedure for posterior mediastinal tumors with respiratory distress. NB; neuroblastoma. MRT; malignant rhabdoid tumor. SMARCA4; smarca4-deficient thoracic sarcoma. ECMO; extracorporeal membrane oxygenation. ☨; died within acute phase

**Fig. 4:**
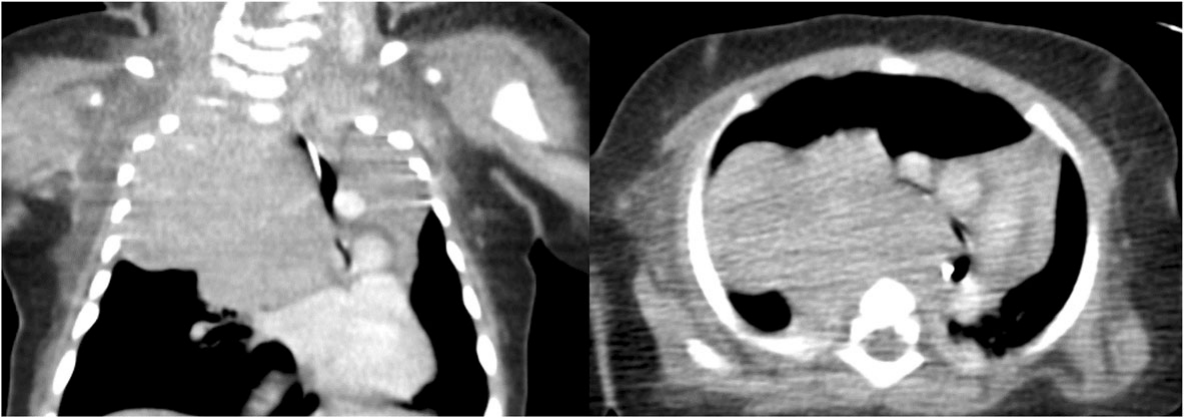
Computed tomography images of a patient undergoing subtotal tumor resection with extracorporeal membrane oxygenation stand-by. The huge posterior mediastinal tumor (neuroblastoma) is compressing the trachea and right main bronchus

Among the five posterior mediastinal tumors, there were three neuroblastomas, one malignant rhabdoid tumor, and one SMARCA4-deficient thoracic sarcoma. Two patients had massive pleural effusion, one had tracheal compression, and two had both pleural effusion and tracheal compression (Fig. 3). Regarding the three patients with tracheal compression, one who showed hypoxemia had < 50% compression and two had ≥50% compression, with initial symptoms of wheezing, tachypnea, hypoxemia, and cardiopulmonary arrest. Respiratory symptoms remained after drainage of the pleural effusion in the patient with ≥50% tracheal compression. The tumor causing tracheal compression required sub-total resection with extracorporeal membrane oxygenation (ECMO) stand-by before chemotherapy could be initiated, (Fig. 4) as tumor necrosis due to chemotherapy can cause tumor enlargement and lead to fatal tracheal compression. In this case, tumor resection was successfully achieved without ECMO. Regarding the two tumors with tracheal compression without pleural effusion, one was diagnosed via subclavian lymph node excisional biopsy under local anesthesia and one was diagnosed solely based on tumor marker findings. Regarding the two cases of pleural effusion without tracheal compression, one was diagnosed by core needle biopsy under local anesthesia and one was initially diagnosed based on tumor marker findings; however, thoracoscopic tissue biopsy was later performed after all courses of chemotherapy had been completed.

Four of the five patients achieved tumor regression and survived the acute phase, while one patient died during this period. One patient with malignant rhabdoid tumor died 15 months after the onset owing to recurrence in the cranium.

#### Clinical course of the patient that died in the acute phase

The patient had originally undergone esophago-esophagostomy for esophageal atresia as a neonate. During the follow-up, she developed gastroesophageal reflux and passage disturbance of the esophagus at 15 years old. Imaging revealed thickening of the esophagus and swelling of the para-esophageal lymph nodes. An excisional biopsy of the subclavian lymph node led to the diagnosis of malignant lymphoma, which was later corrected to SMARCA4-deficient thoracic sarcoma based on genetic examination. The main and right bronchi were compressed by this tumor. Although intubation into the left bronchus temporarily improved the respiratory condition, rapid tumor expansion led to cardiopulmonary arrest, and ECMO was started. Her general condition worsened despite chemotherapy and she died 3 months after the onset of the pulmonary symptoms.

## Discussion

A favorable prognosis in pediatric patients with malignant mediastinal tumors is obtained by appropriate chemotherapy rather than by surgical intervention. Therefore, an efficient and accurate diagnosis of the tumor is imperative to achieve the desired outcome. However, performing biopsies under general anesthesia to arrive at an accurate diagnosis is dangerous in patients with respiratory distress caused by mediastinal tumors, as invasive mechanical ventilation can aggravate airway obstruction by increasing mass effect, and may threaten the life of the patient. Therefore, in order to evaluate safe diagnostic strategies for pediatric patients with malignant mediastinal tumors with respiratory distress, we retrospectively analyzed the records of 12 such patients treated at our hospital.

In 11 of the 12 cases, accurate tumor diagnosis was achieved at our hospital without using measures requiring general anesthesia. Among these 11 cases, two neuroblastoma cases did not involve tumor biopsy and were instead diagnosed by tumor markers. In the one patient who was diagnosed under general anesthesia, the tumor was sub-totally resected with ECMO stand-by, as there was no alternative due to severe tracheal compression caused by the tumor.

**Fig. 5:**
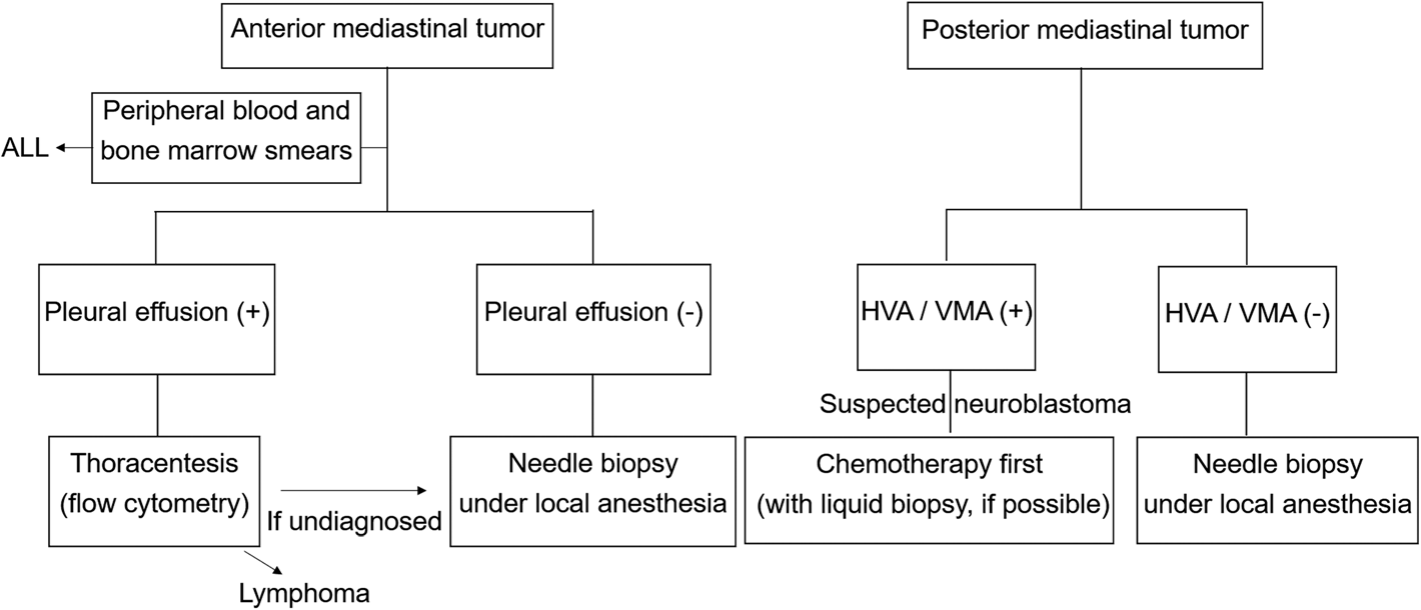
Our diagnostic strategy for anterior and posterior mediastinal tumors with severe respiratory distress. ALL; acute lymphoblastic leukemia. HVA; homovanillic acid. VMA; vanillylmandelic acid

Based on this study, the diagnostic strategy for pediatric malignant mediastinal tumors with respiratory distress was summarized depending on the location of the tumor as shown in Fig. 5. For anterior mediastinal tumors, which mostly include lymphoma, we utilized cytology and flow cytometry of the pleural effusion, if present. If there was no pleural effusion, we performed core needle biopsy under local anesthesia to maintain spontaneous breathing [3]. Acute lymphoblastic leukemia is also noted among anterior mediastinal tumors [2] and is usually suspected based on peripheral blood smear test results. At our hospital, peripheral blood and bone marrow smears are routinely prepared for patients with suspected hematologic malignancy and those with solid tumor requiring evaluation for bone marrow metastases such as neuroblastoma.

In contrast, the majority of posterior mediastinal tumors were neuroblastomas. We can used tumor markers such as homovanillic acid (HVA) and vanillylmandelic acid (VMA) for the diagnosis. As the chemotherapeutic protocol for neuroblastomas varies by risk group, accurate information on the *MYCN* status, pathological status of differentiation, and genetic aberration is necessary to ensure adequate treatment. As neuroblastomas are heterogenetic tumors, a needle biopsy is usually not recommended [4]. Therefore, a thoracoscopic biopsy under careful general anesthesia is desirable if the respiratory condition allows. However, as neuroblastomas arising from the mediastinum are generally known to have a better prognosis than those arising from the adrenal glands [5], chemotherapy may be performed when the respiratory condition is too severe for general anesthesia (Fig. 5). In such cases, a liquid biopsy may provide useful information on factors such as the *MYCN* status and genetic aberration [6, 7]. In the future, multi-gene panel testing, which searches for mutations in several genes simultaneously using next-generation sequencing technology, will be added for pediatric oncology/hematology patients including those with similar cases at our hospital. When neuroblastoma, which can enlarge during chemotherapy due to necrosis or tumor bleeding, causes severe respiratory distress [8], partial resection is of little value for saving the patient’s life. We must adopt a “Wait and See” approach under ECMO until chemotherapy and interventional radiology show an effect on tumor regression and hemostasis [9]. When the neuroblastoma does not originate from the mediastinum but rather develops into the mediastinum from the retroperitoneum, the prognosis is usually poorer than when it originates from the mediastinum [5], and careful observation is required.

If HVA and VMA are negative in posterior mediastinal tumors, rarer tumors, such as germ cell tumor, liposarcoma, and SMARCA4-deficient thoracic sarcoma should be considered in the differential diagnosis [10]. In such cases, a biopsy must be performed by any means necessary. If a needle biopsy is possible, it should be a priority. However, when imaging examinations suggest that a needle biopsy is too difficult to safely pursue, a thoracoscopic or open biopsy under general anesthesia should be considered. Blank et al. proposed risk stratification strategies for anesthetic management [11]. No serious complications are expected in patients at low risk; asymptomatic or mildly symptomatic, without postural symptoms or radiographic evidence of significant compression of structures. However, in intermediate-; mild to moderate postural symptoms, tracheal compression < 50% and for those at high-risk with severe postural symptoms, stridor, cyanosis, tracheal compression > 50% or tracheal compression with associated bronchial compression, pericardial effusion or in those with SVC syndrome, individualized management approaches are necessary. During anesthesia, the maintenance of spontaneous respiratory effort is strongly recommended by several studies in order to avoid the risk of airway collapse [11, 12]. In high-risk patients in particular, preparation for the use of cardiopulmonary bypass or ECMO is also recommended [11]. With these diagnostic strategies, we were able to obtain satisfactory outcomes.

There are a few limitations associated with our study. First, the patients displaying tumor-related respiratory distress were evaluated retrospectively from the available medical records. A prospective study would therefore be desirable. Second, as the number of cases was limited, a thorough evaluation with a larger sample size should be performed in the future.

## Conclusions

We reviewed our 10-year experience with patients who had developed respiratory symptoms due to malignant mediastinal tumors, with a focus on the diagnostic approach. In 11 of 12 cases, safe and accurate diagnosis was achieved using methods that did not require general anesthesia, thus eliminating the additional risk of increased respiratory distress or respiratory collapse. Our review confirmed that our diagnostic strategy, that considered the tumor location, was both safe and effective in achieving a good prognosis for the patients involved.

## Data Availability

The datasets generated and/or analyzed during the current study are available from the corresponding author on reasonable request.

## Abbreviations

ECMO: Extracorporeal membrane oxygenation
HVA: Homovanillic acid
VMA: Vanillylmandelic acid
